# How to survive a periviable birth baby with birth weight of 450g: A case report

**DOI:** 10.1097/MD.0000000000031356

**Published:** 2022-10-21

**Authors:** Binzhi Tang, Qiying Ling, Qian Yang, Maojun Li, Wei Shi, Qing Wu

**Affiliations:** a Department of Pediatrics, Sichuan Academy of Medical Sciences & Sichuan Provincial People’s Hospital, Chengdu, China; b Department of Pediatrics, Clinical College of University of Electronic Science and Technology of China, Chengdu, Sichuan Province, China.

**Keywords:** case report, extremely low birth weight, individualized management, periviable birth, survive

## Abstract

An increasing number of periviable birth newborns (PVBs) have emerged with concurrent growing high-risk pregnancy. To date, postnatal management of PVBs remains one of the most challenging issues and limited studies have been reported.

**Patient concerns:**

A female baby born at 23^0/7^ weeks of gestation with birth weight of 450g.

**Diagnosis:**

PVB baby, respiratory distress syndrome (RDS), ventilator associated pneumonia (VAP), intraventricular hemorrhage (IVH), metabolic bone disease of prematurity (MBDP), transient hypothyroxinemia of prematurity (THOP), bronchopulmonary dysplasia (BPD) and retinopathy of prematurity (ROP).

**Interventions:**

Individualized treatment and intensive care, including neonatal resuscitation, effective respiratory and circulatory support, venous access and nutrition, prevention and treatment of infection, management of endocrine and metabolic problems, individualized nursing such as developmental supportive care, integrated oral motor interventions, skin care, family-integrated-care, etc were performed according to existing literature.

**Outcomes:**

The baby was discharged home after 138 days of hospitalization with body weight of 2700 g, a full oral feed achieved, and without any requirement of respiratory support or oxygen supply. Now she is 38-month-old, with no significant long-term adverse sequelae.

**Lessons:**

Our case expands the experience and knowledges of individualized and intensive management of PVB babies in their early life days, which increase PVBs’ survival and improves their prognosis.

## 1. Introduction

Periviable birth (PVB) neonates refer to mothers deliver at a periviable (20^0/7^~25^6/7^ weeks) gestation.^[1]^ PVB has been implicated in a higher mortality and morbidity of complications including respiratory distress syndrome (RDS), ventilator associated pneumonia (VAP), intraventricular hemorrhage (IVH), bronchopulmonary dysplasia (BPD) and retinopathy of prematurity (ROP). Improving survival in PVB babies mandates improved evidence-based practices. We present herein early management of a PVB girl with gestational age (GA) 23^0/7^ weeks and birth weight (BW) 450g, and compare our results to published data.

## 2. Case report

**Figure 1.**
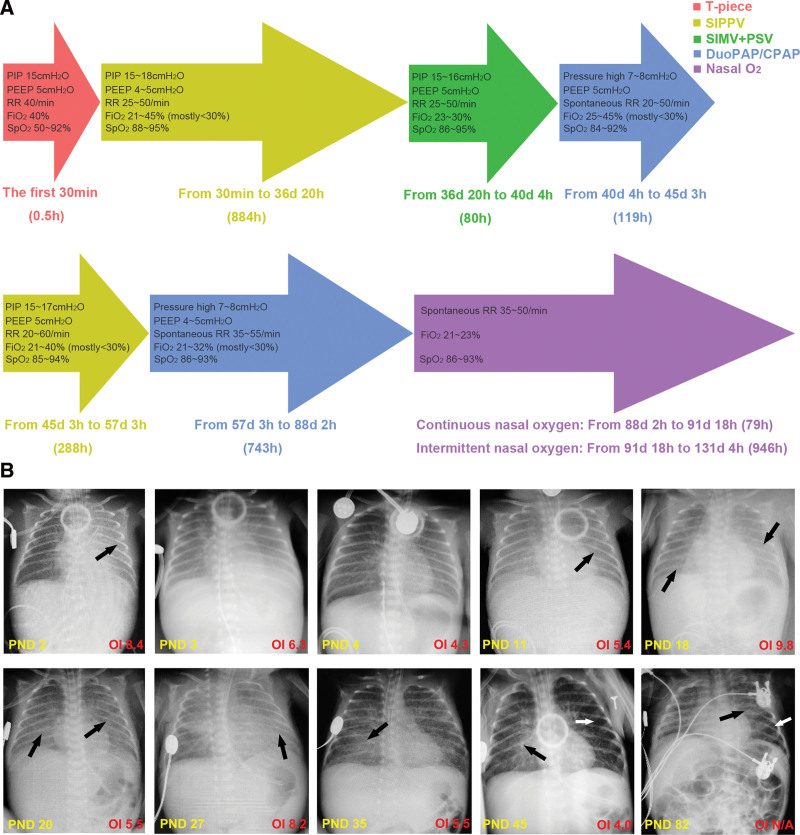
Respiratory support and pulmonary disorders. (A) Modes, parameters and duration of respiratory support in NICU; (B) CXR images and OI on different PNDs. Atelectasis at PND 2 (black arrow) suggested RDS. Increased transparency and decreased OI were seen after PS and MV (PND 3 and 4). VAP or BPD was presented by generalized opacification at PND 11, 18, 20, and 27, streaky areas opacification at PND 35, areas of patchy opacification (black arrows) and over-inflation (white arrows) at PND 45 and 82. BPD = bronchopulmonary dysplasia, CXR = chest X ray, MV = mechanical ventilation, NICU = neonatal intensive care unit, OI = oxygenation index, PND = postnatal day, PS = pulmonary surfactant, RDS = respiratory distress syndrome, VAP = ventilator associated pneumonia.

Perinatal strategies: Antenatal corticosteroid was given once 3 hour before emergency vaginal delivery due to inevitable abortion. She appeared cyanosis and soft, no breath, and a heart rate (HR) of 40 beats/min at birth. Following guideline,^[2]^ she was quickly moved to a preheating radiant warmer at 35^◦^C, immediately intubated and connected to a T-piece resuscitator for positive-pressure ventilation (Fig. 1A), accompanied by chest compressions. An umbilical vein catheter (UVC) was inserted and epinephrine, saline and sodium bicarbonate were injected. Subsequently, 200 mg/kg of pulmonary surfactant (PS) was given and HR elevated to 150 beats/min and saturation of pulse oxygen (SpO_2_) to 90% to 92% at 10 minutes of age. The body was wrapped with polythene film once, then wet film quickly removed and wrapped again with new film. Body temperature (BT) was 36.1°C before transferred to neonatal intensive care unit (NICU). Physical examination at NICU admission was: lethargy, weak pulses and cooling extremities, BT 35.5^◦^C, HR 150 beats/min, artificial breath 41 times/min, and SpO_2_ 87%, respectively.

Respiratory support and ventilator/oxygen related complications: The baby received 2114 hour of Mechanical ventilation (MV) in total, including invasive MV for 1252 hour, noninvasive MV for 862 hours, and nasal oxygen inhalation for 79 hours, then completely independent of ventilator/oxygen at postnatal day (PND) 131 or post menstrual age (PMA) 41^4/7^ weeks (Fig. 1A). Respiratory support was adjusted according to real-time breath, blood gas or transcutaneous oxygen/carbon dioxide pressure (transcutaneous oxygen pressure/transcutaneous carbon dioxide pressure), and chest X-ray (Fig. 1B). 200 mg/kg of PS was given at 5 minutes, 6 and 52 hours, respectively, after birth. A lung protective strategy was adopted, that is, to maintain arterial oxygen pressure 50 to 80 mm Hg, arterial carbon dioxide pressure (PaCO_2_) 30 to 70 mm Hg, and SpO_2_ 88% to 94% with lowest ventilatory parameters (Fig. 1A), in order to avoid lung injury or ROP caused by unduly high airway pressure or fraction of inspired oxygen (FiO_2_). Other beneficial strategies include: endotracheal suctioning using a closed suction device; kept in the left lateral, the prone and the supine position alternately, and a dorsal elevated position (15°). Despite of physical stimulation and caffeine, frequent apneic episodes existed during the first noninvasive MV period (PND 40~45, Fig. 1A) and intubated again at PND 45. 12 days later, the second extubation attempt succeed. Moreover, from PND 60 or PMA 31^4/7^ weeks on, the parents entered NICU for family-integrated-care (FIcare) including kangaroo care and feeding. With these efforts, completely spontaneous breath and oxygen independence were achieved at PND 131 (Fig. 1A). A modified corticosteroid regimen was adopted for BPD treatment: 1.5 mg/kg of hydrocortisone administrated intravenously q6h from PND 37 to 41, gradually weaned until complete withdrawal at PND 77. ROP of stage 2 was found in zone 2 of retina by her first examination at PND 63 or PMA 32^0/7^ weeks. Fortunately, ROP gradually resolved and did not affect her vision after intravitreal injection of ranibizumab at PMA 32^6/7^ weeks.

**Figure 2.**
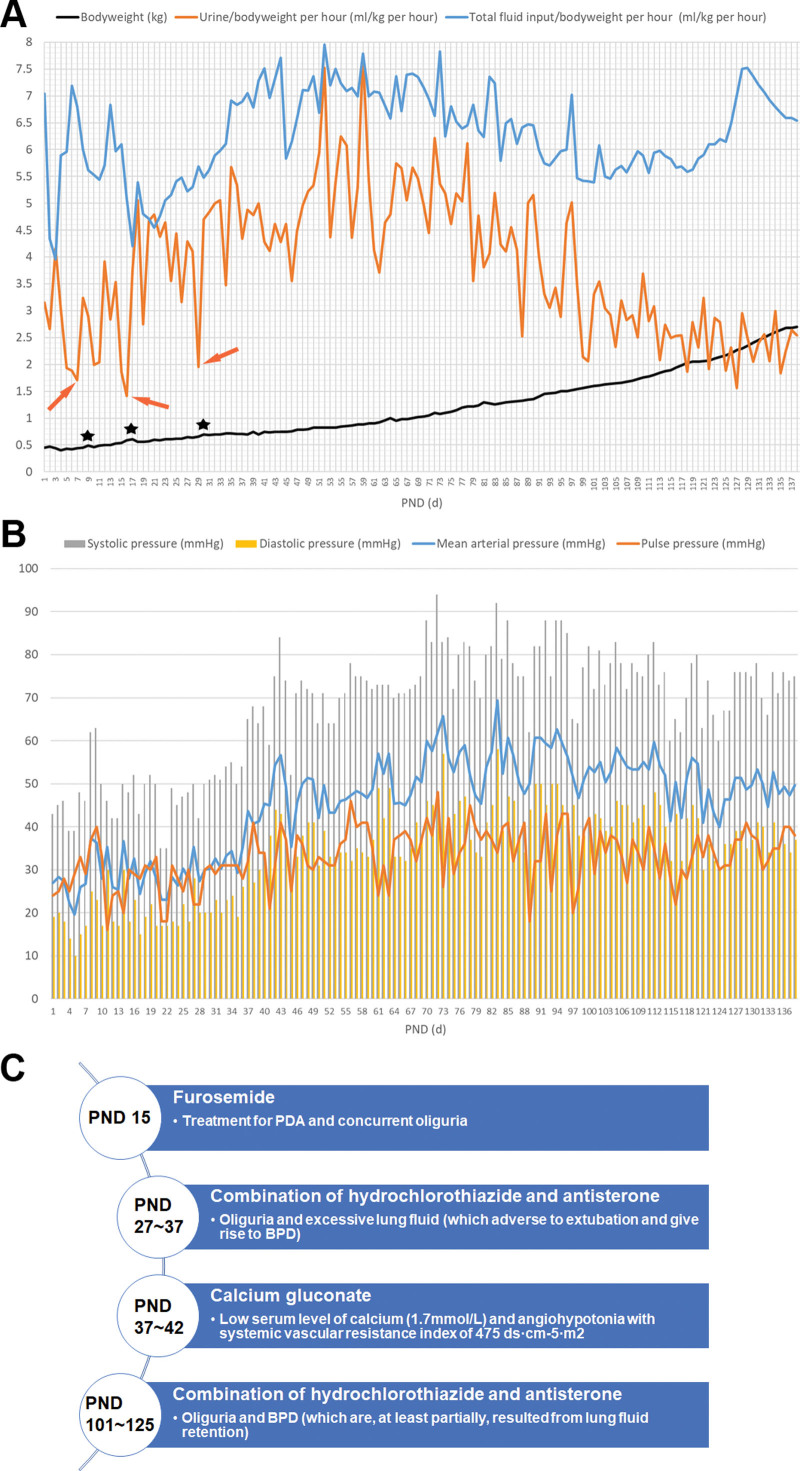
Circulatory management of the PVB baby. (A) Fluid balance and body weight growth curve. A consequence of sudden increase of weight gain accompanied by edema (black asterisks) was likely associated with oliguria (orange arrows) in early PNDs; (B) Integrated trend of blood pressure; (C) Administration of diuretics or vasoactive drugs, the time of drug administration were demonstrated in the circle, while the drugs and the reason for drug administration demonstrated in the rectangle. PND = postnatal day, PVB = periviable birth.

Circulatory support: Fluids were initiated at 100 mL/kg/day and regulated according to fluid balance and body weight. The lowest bodyweight of 400 g was found at PND 4 due to postnatal dehydration, following by oliguria (<2 mL/kg/h) episodes (Fig. 2A). Besides, mean arterial pressure was even lower than pulse pressure at PND 3 to 10 and PND 15 to 20, and lowest diastolic pressure was only 10 mm Hg (Fig. 2B), describing an unsatisfactory circulation.10 mg/kg of oral ibuprofen was attempted at PND 15 to close a patent ductus arteriosus (PDA) with 1.7 mm of width, but only once due to feeding intolerance (FI), meanwhile furosemide was given (Fig. 2C). PDA was found completely closed at PND 127. 2 to 3 g/kg of human albumin was given at PND 6, 13, 73, and 96, respectively, when serum albumin <29.0 g/L. 0.1 to 0.133 IU/kg of red blood cell suspension was given at PND 6, 13, 18, 34, 44, 59, 77, and 96, respectively, when hemoglobin <100 g/L, especially when MV/oxygen dependent.

Neurodevelopmental supportive strategies: To prevent the developing brain from damages such as IVH, a developmental supportive care (DSC) was provided, including: protecting skin: polythene film was used to make body dry at postnatal resuscitation, and hydrocolloid pad was covered on the skin before adhesive tapes or electrode patches attached; minimal handling to keep incubator temperature and humidity and reduce iatrogenic infection; reducing light stimulation; environmental noise <50 dB; ventilator adjustments mainly according to transcutaneous oxygen pressure/transcutaneous carbon dioxide pressure rather than blood gas sampling to reduce pain, iatrogenic anemia and infection. Besides routine postnatal vitamin K1, etamsylate and hemocoagulase were also used to prevent IVH. Moreover, phenobarbital, midazolam, and fentanyl were given during MV period as needed. Owing to DSC, hemostatics and analgesics-sedatives, no major brain injury such as sever IVH (grade III/IV) occurred. Caffeine citrate was the only nervous stimulant prescribed, which daily administrated from PND 5 to PND 111.

Anti-infective therapy and prevention of infection: A detailed antibiotic regimen was presented in Table 1, and fluconazole, a typical antimycotic drug, was empirically used from PND 9 to PND 41. Besides, intravenous immunoglobulin (IVIG) was given at PND 52 to facilitate anti-infection. The baby was separated in a single ward with a continuous air purifier, only designated medical staff and the parents were allowed entering and aseptic procedures strictly obeyed.

**Table 1:** Intravenous administration of antibiotics.

PND (d)	Antibiotics	Dose	Reasons for initiating antibiotic therapy
1~2	Mezlocillin/sulbactam	50 mg/kg, q12h	Empiric antibiotic therapy of gram-negative bacteria infection
2~12	Combination of meropenem and penicillin	Meropenem:15 mg/kg, qd Penicillin: 100,000 IU/kg, q12h	Unpleasant blood gas result (pH 7.12, BE -12.4 mmol/l, Lac 5.9 mmol/l) and elevated serum PCT level (35.5 ng/mL), drastically decreased WBC, neutrophil and platelet count (Fig. 3A and B) and unfavorable CXR image (Fig. 1B).
12~16	Penicillin	100,000 IU/kg, q12h	Low level of procalcitonin (0.4 ng/mL) and CRP (0.7 mg/L), and downregulated ventilatory parameters.
16~24	Combination of meropenem and penicillin	Meropenem:15 mg/kg, qd Penicillin: 100,000 IU/kg, q12h	Yellow sputum with fluctuant WBC and neutrophil count and elevated CRP (Fig. 3A), accompanied by unfavorable CXR image with high OI (Fig. 1B)
24~42	Cefuroxime	15 mg/kg, q12h	A better blood gas result (pH 7.25, BE -2.6 mmol/l, Lac 0.8 mmol/l) and normal WBC, neutrophil and CRP (Fig. 3A)
45~49	Meropenem	15 mg/kg, q12h	Excessive sputum and frequent apnea that required invasive MV again. Increased WBC and neutrophil count, and elevated CRP (Fig. 3A)
49~52	Cefuroxime	30 mg/kg, q12h	A favorable blood gas result (pH 7.33, BE 1.5 mmol/l, Lac 1.9 mmol/l) and normal WBC, neutrophil and CRP (Fig. 3A)
52~91	Ceftazidime	30 mg/kg, q8h	Frequent apnea and lung rales, and elevated CRP (Fig. 3A)

BE = base excess, CRP = C-reaction protein, OI = oxygenation index, WBC = white blood cell.

Central venous access, feeding and nutrient supply: UVC served as the only venous access until its removal at PND 18. Meanwhile, a peripherally inserted central catheter was inserted through the right basilic vein, and consecutively retained till PND 92 when parenteral nutrition (PN) no longer needed. PN was prescribed as follows: protein was initiated at 2.0 g/kg/d at PND 2 and increased up to 2.5 to 3.0 g/kg/d. Carbohydrates were given as 5% to 16% glucose since PND 1, with glucose infusion rate at 4.0 to 8.0 mg/kg/min. Lipids were given as 20% intralipid, starting at 2.0 g/kg/d since PND 3 and increased up to 3.0 to 3.5 g/kg/d. Total calorie was started at 55 kcal/kg/d at PND 2, gradually increased to 100 kcal/kg/d at 2 weeks, and reached 120 kcal/kg/d at 6 weeks after birth. An elevated alanine aminotransferase (89 U/L) was found at PND 34 and dropped to 24 U/L at PND 38, suggesting a mild and temporary hepatic dysfunction. Meanwhile elevated serum level of conjugated bilirubin (49.9 μmol/L at PND 38 and 35.7 μmol/L at PND 52) and total bile acid (25.7 μmol/L at PND 43 and 29.7 μmol/L at PND 52) appeared, describing cholestasis. Then, hepatic protectants such as ursodeoxycholic acid, ademetionine 1,4-butanedisulfonate and diisopropylamine dichloroacetate were given, and results were encouraging (alanine aminotransferase 14 U/L, conjugated bilirubin 7.5 μmol/L, and total bile acid 12.2 μmol/L, at PND 77). Oral smear with breastmilk was started at PND 1, then minimal enteral feeding of breastmilk through oral-gastric tube was initiated and feeding amount increased following guidelines.^[3]^ Human milk fortifier (HMF) was added at PND 57 when breastfeeding reached 100 ml/kg/d. Integrated oral motor interventions (IOMIs) were initiated since PND 76 or PMA 33^6/7^ weeks to enhance suck-swallow-breathe coordination. Oral feeds were started since PND 85 or PMA35^1/7^ weeks, and full oral feeds (FOF) achieved at PND 108 or PMA 38^3/7^ weeks.

Management of endocrine and metabolic problems: Blood glucose fluctuated at a high level (4.6~14.8 mmol/L) in the first 2 weeks despite restricted glucose infusion rate, and 0.01 to 0.15 IU/kg/h of insulin was administrated when blood glucose over 10.0 mmol/L. Supplementary calcium gluconate, sodium glycerophosphate and vitamin D3 were initiated at PND 2, PND 9, and PND 41, respectively. However, decreased serum levels of phosphate (0.8~1.4 mmol/L) accompanied with increased serum levels of alkaline phosphatase (ALP, 411~710 IU/L) persisted in the first 3 months, suggesting a possible metabolic bone disease of prematurity (MBDP). A decreased total tetraiodothyronine (TT4, 20.9 nmol/L) level and a normal thyroid stimulating hormone (TSH, 1.9 mIU/L) level were detected at PND 14. 3 μg/kg of levothyroxine was daily administrated from PND 14 to PND 69. TT4 of 100.5 nmol/L and TSH of 3.2 mIU/L were found at PND 83.

The baby was discharged home after 138 days of hospitalization with body weight of 2700 g and without any requirement of respiratory support or oxygen supply, and a FOF of 53 mL milk was given every 3 hours. Now she is 38-month-old, with body weight of 9600 g and height of 83 cm, and recent audiometry and retinoscope examination showed no abnormities. She can walk, run, jump, and ride a bicycle, and communicate to families and friends, describing a well motor and intelligence development.

### 2.1. Informed consent

Written informed consent was obtained from the patient for publication of this case report and any accompanying images.

## 3. Discussion

The percentage of infants born before 24 weeks of gestation who survived without neurodevelopmental impairment was approximate 20%,^[4]^ describing challenges in management of the PVBs. Experience and considerations from this case and related literature are discussed and summarized as following:

Individualized postnatal resuscitation and warm keeping: considering high risk of hyperoxia-induced BPD or ROP in PVB babies, FiO_2_ remained <40% even advanced cardiopulmonary resuscitation required. Make body dry with polythene wrap to prevent towel-induced skin injury. Warmth and moisture maintenance was emphasized to avoid hypothermia, dehydration, scleredema and circulatory disorders.^[5]^ 0.6°C drop of BT demonstrated a well warm keeping during transport. Besides, setting incubator temperature at 34°C to 35°C and humidity at 85% to 95% in the first month, wrapping body entirely and minimal handling to avoid frequent incubator opening, were all beneficial to maintain BT at 36.5°C to 37.5°C as required.^[2]^

Individualized DSC: soft silicone hydrocolloidal dressings were used to prevent sticky materials induced skin damage, as the reported benefits to facilitate atraumatic and non-painful adhesive dressing removal.^[6]^ Indeed, DSC effectively maintained skin integrity, minimizing stress and pain, promoting comfort and safeguarding sleep, and thus reducing brain injury. These results agreed with previous study that DSC provides a healing environment to maximize safe and comfort whilst minimizing potential harms.^[7]^

Effective respiratory support: following guidelines,^[8]^ rescue PS was early administrated when intubation was required. Besides, targeting lower SpO_2_ (88%~92%) may be more appropriate for PVB babies in early PNDs.^[9]^ Considering the reported benefits,^[10]^ a lung protective strategy was implemented to maintain lung recruitment and appropriate arterial oxygen pressure/PaCO_2_, and also to reduce complications associated with hyperoxia and airway pressure/volume injury, facilitating an early oxygen independence.^[10]^ Unnecessary endotracheal suctioning should be avoided as it could lead to disturbances of cerebral hemodynamics, and a closed suction device was preferentially considered to prevent alveolar collapse and cross-infection.^[11]^ In addition, the left lateral position and the prone position were used as alternatives to supine position, as left lateral or prone position may be related to a more efficient breathing pattern through reducing gastroesophageal reflux (GOR) and apnea, and increasing thoracoabdominal synchrony.^[12]^ Caffeine was initiated early as it has been evidenced to facilitate extubation with reduction in apneic episodes and BPD, and better neurodevelopmental outcomes.^[8]^ Of notice, the unsuccess of extubation attempt at PND 45 was more likely attributed to frequent apnea rather than pulmonary incompetency (Fig. 1A and B). Permissive hypercarbia was another strategy adopted, since moderate elevation of PaCO_2_ (60~75 mm Hg) in early life days and during MV weaning seemed to shorten duration of MV without adverse sequelae in extremely low birth weight babies.^[8]^

Intensive circulation management: episodes of unstable blood pressure, oliguria/edema, metabolic acidosis, dyspnea and FI in the first month were possibly related to hypovolaemia, angiohypotonia, myocardial dysfunction and PDA (Fig. 2A and B). Saline boluses, blood product and calcium gluconate were prescribed as antihypotensive therapy;^[13]^ meanwhile restricted fluids or diuretics, and ibuprofen were given to promote PDA closure according to cochrane data.^[14]^

**Figure 3.**
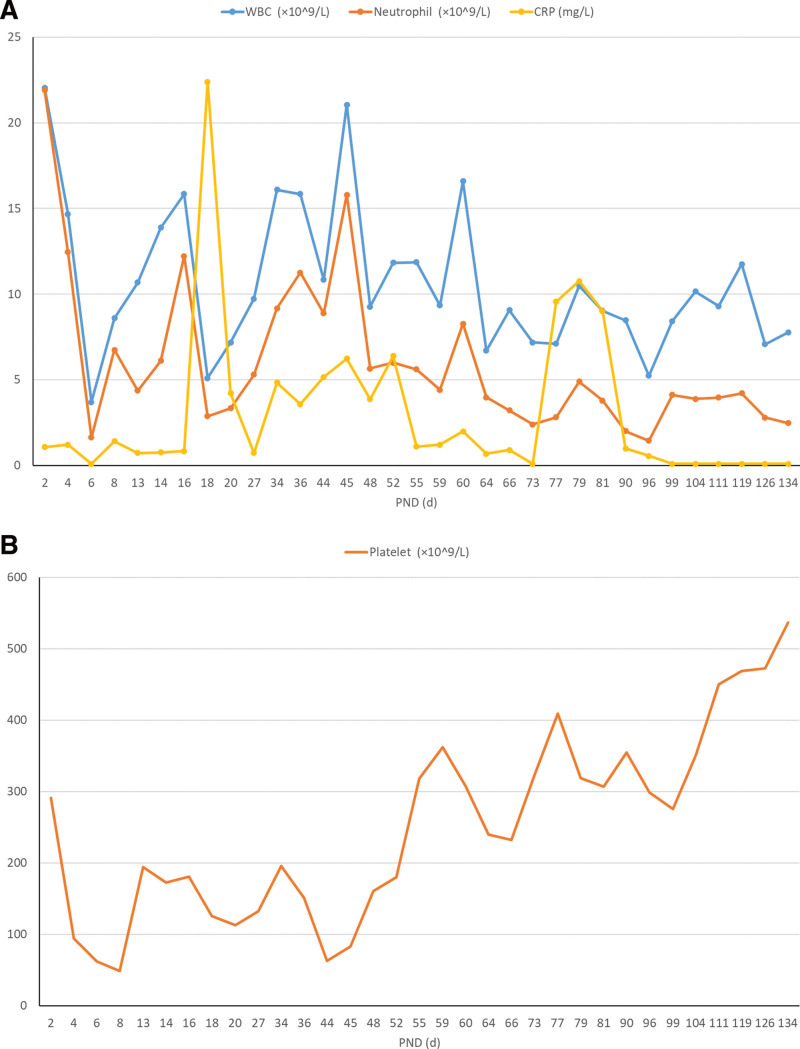
Main results of infectious indicators. (A) WBC and neutrophil count, together with CRP; (B) platelet count. Note the significant consumption of WBC, neutrophil and platelet in early PNDs, suggesting a severe infection; whereas no CRP elevated correspondingly due to the extremely premature immune system. CRP = C-reaction protein, PND = postnatal day, WBC = white blood cell.

Targeted anti-infective strategies and hygiene: PVBs were generally associated with perinatal infections, especially gram-negative bacteria.^[15]^ Empirical antibiotics is a key to successful infection control. IVIG was also prescribed when signs of infection remained despite a prolonged antibiotic use, as episodes of infection in preterm infants were reportedly reduced by IVIG.^[16]^ The reason for fungal prophylaxis was: a consecutive use of broad-spectrum antibiotics, immunocompromise particularly when systemic steroid given, and low platelet count (49 × 10^9^/L) at PND 8 (Fig. 3B). A protective hygiene was strictly followed as PVB babies are susceptible to nosocomial infections.^[17]^ Even though, recurrent infections were observed respectively at PND 16, 45, and 52, which may be attributed to immunocompromise of the baby, lack of laminar air-flow and VAP.

Optimize nutritional support and consecutive breast milk: PVB babies were easily suffered from nutritious deficiency and positive nutritional strategies were recommended to improve growth outcomes.^[18]^ UVC or peripherally inserted central catheter provides an ideal vein tunnel for PN,^[19]^ and no catheter-related complications occurred during 3 months of central venous access. PN was daily prescribed and adjustments individualized according to fluid balance, and serum levels of glucose, triglyceride, protein/nitrogen and electrolytes. Hepatic dysfunction and cholestasis were mainly attributed to prolonged PN, as no evidence of hepatotropic virus infection were found. Therefore, positive enteral feeding and hepatic protectants were given to alleviate PN-induced liver impairment. Oropharyngeal colostrum was early initiated and continued throughout the whole orogastric gavage period as it could effectively reduce VAP, FI, and enterocolitis.^[20]^ Besides, minimal enteral feeding of breast milk was positively given to promote intestinal flora and immunity, improve gut motility, and achieve FOF earlier.^[21]^ Approximately 13 g/kg/d of bodyweight gain was observed in the first 2 months, and the speed increased to approximately 17 g/kg/d after supplementary HMF added (Fig. 2A), which was consistent with previous study showing exclusive breastfeeding would result in slower postnatal growth.^[22]^

Prevention and treatment of endocrine and metabolic problems: transient hyperglycemia and insulin requirement in early PNDs of the PVB baby may be attributed to excess glucose intake, pain/stress, infection, steroids, low phosphatemia, and glucose intolerance commonly seen in extremely preterm infants.^[23]^ MBDP is common in extremely low birth weight neonates particularly if exclusively breast fed.^[24]^ Despite of supplementary calcium, phosphate, vitamin D3 and HMF, a mild MBDP without any radiographic changes, was suggested by elevated ALP, persistent hypophosphatemia, MV dependence and extrauterine growth retardation (both body weight and height are below the 10th percentile of girls of the same PMA). Transient hypothyroxinemia of prematurity (THOP) was reportedly observed in up to 50% of extremely preterm infants.^[25]^ Since the parents had no thyroid disease, and TT4 level elevated by minimum dose of levothyroxine, it is reasonable to consider the decreased TT4 level in the PVB baby as a THOP, rather than congenital hypothyroidism. Although influences of THOP in preterm infants are controversial,^[25]^ supplementary levothyroxine in extremely preterm babies are generally required,^[26]^ and aiming of intervention is to maintain a favorable prognostic outcome with minimized TSH inhibition,^[25]^ as achieved in this case.

FIcare and IOMIs: FIcare including kangaroo care was initiated soon after extubation, as kangaroo care has been reported to facilitate cardiorespiratory stability and oxygenation, and improve breastfeeding in premature infants.^[27,28]^ Prolonged stay in the NICU usually raises mothers’ anxiety and even results in lactation reduction.^[29]^ In this case, the mother’s score of anxiety was significantly reduced by FIcare (severe anxiety before FIcare, mild anxiety after 1 month of FIcare, and no anxiety after 2 months of FIcare), which was consistent with previous studies.^[30]^ Moreover, parents can learn knowledges and skills during FIcare, beneficial to a better care after discharged home. Interestingly, all the recurrent infections happened before initiation of FIcare, which agreed with previous studies, that FIcare did not increase risk for infection^[31]^; conversely, mother-baby skin contact and breastfeeding contribute to stronger immunity. IOMIs has been evidenced to improve feeding performance without increasing related complications.^[32]^ As described above, 32 days was spent from the beginning of IOMIs to successful FOF for this PVB baby. In contrast, results from a multicenter randomized controlled study showed that in preterm infants born 26 to 30 weeks gestation, oral training initiated at PMA 30^0/7^ to 32^6/7^ weeks, oral feeds initiated at PMA 32^0/7^ to 32^6/7^ weeks, and FOF achieved at PMA 36^0/7^ to 36^6/7^ weeks, indicating 23 days of duration from initiation of training to FOF.^[33]^ All these results suggested a delayed initiation of both oral training and oral feeds, and also a longer training period before reaching FOF in PVB baby, compared to those with GA of 26^0/7^ to 30^6/7^ weeks. This may be attributed to the very immature oral-pharyngeal neuromuscular system in PVB babies and thus a longer maturational processes to achieve coordinated suck-swallow breathe is required.

Management of complications: antenatal steroid reportedly reduced risk/severity of RDS if full course was given.^[34]^ In this case, RDS inevitably occurred due to insufficient antenatal corticosteroid and also the immature lung. Lung recruitment and decreased OI were seen at PND 4 after therapeutic PS and MV (Fig. 1B). A similar situation was also observed at PND 35 or PMA 28^0/7^, which may be attributed to the endogenous surfactant production along with marked diuresis (Fig. 2A). Despite of protective strategies including strict hygiene, closing PDA and preventing GOR, and immunoenhancement like IVIG and breastfeeding, recurrent VAP occurred. Any endeavors to reduce MV dependence, like lung protective ventilation and caffeine are beneficial as prolonged MV is the primary contributor to VAP.^[35]^ Incidence of IVH is increasing with decreased GA and BW, and moderate/severe neurodisability ensues if sever IVH occurs.^[36]^ Fortunately, only asymptomatic IVH (Grade I) was detected on early screening ultrasound owing to protective measures as described above. Clinical manifestations of BPD were MV/oxygen dependence, dyspnea, apnea, respiratory secretions/bronchospasms and typical radiographs (Fig. 1B).^[37]^ Dexamethasone seemed to reduce the incidence of BPD, but was associated with neurodevelopmental impairment.^[38]^ Therefore, hydrocortisone was used as it reportedly reduced BPD with improved neurological outcomes in infants born less than 25 weeks’ gestation.^[39]^ Besides, lung protective ventilation, optimized nutrition supply, restricted fluids, diuretics, caffeine, anti-infection, concurrent treatment for PDA, RDS, and GOR, are all helpful in management of BPD.^[40]^ With these efforts, the baby was discharged without any respiratory support, and also no significant ROP occurred owing to restricted FiO_2_.

An increasing number of PVB newborns have emerged in recent years with concurrent growing high-risk pregnancy, and the risk of disability and mortality rate are still very high in PVB infants with BW <500 g. This is till now the first PVB infant born at 23 weeks’ gestation with birth weight of 450 g in southwest China, who successfully survived without any long-term adverse sequelae. Individualized and intensive neonatal management increase survival of PVB babies and improves their prognosis. Experience from this case, together with knowledges from literature reviews, will provide valuable information and guidance for improving early management of PVBs with such low BW.

## Author contributions

Binzhi Tang, Qiying Ling, Qian Yang, Maojun Li, Wei Shi and Qing Wu designed the report; Binzhi Tang, Qiying Ling, Qian Yang, and Qing Wu collected the patients’ clinical date; Binzhi Tang, Qiying Ling, and Qing Wu wrote the paper.

**Conceptualization:** Binzhi Tang, Qiying Ling, Qian Yang, Maojun Li, Wei Shi, Qing Wu.

**Data curation:** Binzhi Tang, Qiying Ling, Qian Yang, Qing Wu.

**Investigation:** Binzhi Tang.

**Methodology:** Maojun Li.

**Software:** Binzhi Tang.

**Writing – original draft:** Binzhi Tang, Qiying Ling, Qing Wu.

**Writing – review & editing:** Binzhi Tang, Qing Wu.
